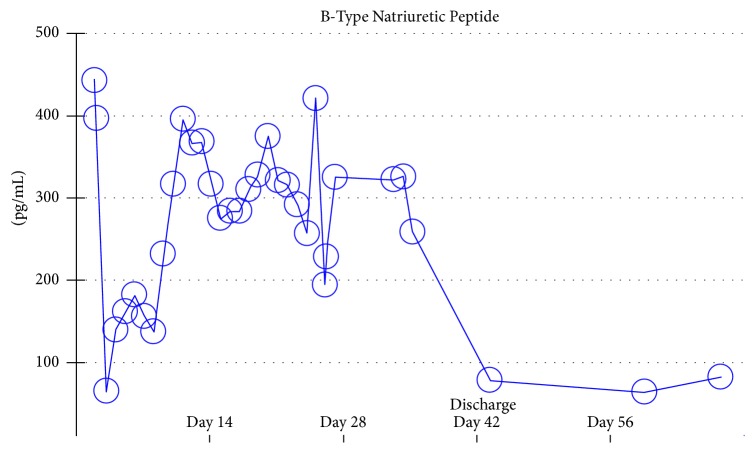# Respiratory Syncytial Virus Associated Myocarditis Requiring Venoarterial Extracorporeal Membrane Oxygenation

**DOI:** 10.1155/2017/7074508

**Published:** 2017-05-08

**Authors:** Anamaria Milas, Aditya Shah, Neesha Anand, Meghan Saunders-Kurban, Samir Patel

**Affiliations:** Advocate Christ Medical Center, Oak Lawn, IL 60453, USA

## Abstract

Severe fulminant myocarditis causing cardiogenic shock can be a rapidly progressing, life threatening condition. Respiratory syncytial virus (RSV) is a very rare infectious culprit infrequently described in medical literature as a cause of myocarditis, particularly in adults. We present a case of acute fulminant myocarditis in a patient with PCR positive RSV infection requiring venoarterial extracorporeal membrane oxygenation (VA-ECMO).

## 1. Background

Cardiogenic shock and heart failure impose significant morbidity and mortality in patients with fulminant myocarditis. When conventional supportive care fails, mechanical circulatory assistance is often required. Viral infections remain one of the most frequent causes of myocarditis, especially in the pediatric population. Common viruses associated with infection include adenovirus, coxsackievirus, parvovirus B19, and influenza [1]. Respiratory syncytial virus (RSV) is seldom implicated as a cause of viral myocarditis in either pediatric or adult populations [2]. After a thorough PubMed literature search using keywords RSV, viral myocarditis, ECMO, and myocarditis, one case documenting RSV associated acute myocarditis in adults was found, requiring peripheral VA-ECMO [3]. We present a unique case of fulminant myocarditis in an adult patient with RSV positive serology leading to cardiogenic shock requiring circulatory support with central VA-ECMO.

## 2. Case

Our patient was a 40-year-old African American male with a past medical history of diabetes mellitus, hypertension, and hyperlipidemia who was in his normal state of health prior to hospital admission. According to the patient's family, he recently traveled from Las Vegas and upon returning abruptly developed fevers, chills, and worsening dyspnea two days prior to presentation. Per history, he had no prior cardiac or pulmonary disorders. He had excellent performance status including vigorous daily physical activity prior to his hospitalization and was compliant with his oral medications. Family denied any recent sick contacts or prior hospitalizations. The patient smoked for 20 years with cessation 6 months prior to presentation. He drank alcohol socially and did not use illicit drugs. Initial vital signs revealed a temperature of 39.5° Celsius, heart rate of 130 beats per minute, blood pressure of 156/101 millimeters of mercury (mmHg), respiratory rate of 30 breaths per minute, and oxygen saturation 92% on room air. On physical exam, the patient had evidence of accessory muscle use with crackles at the lung bases bilaterally. He was tachycardic but no murmur or gallop was noted. His extremities were cool to touch without edema bilaterally.

**Figure 1:**
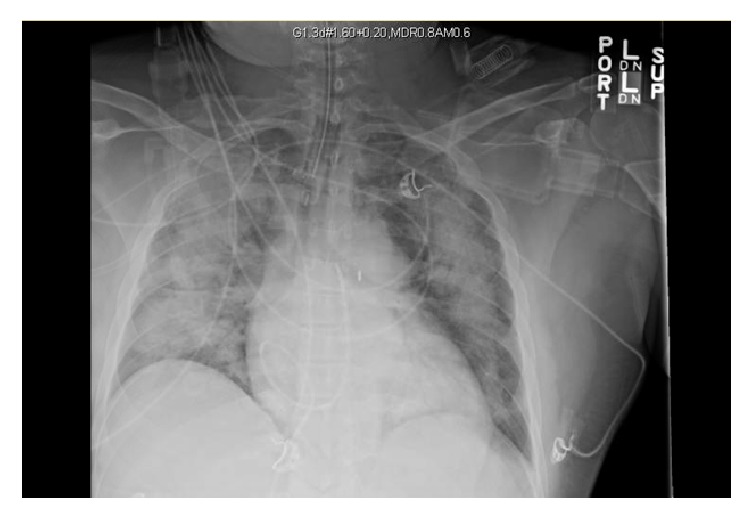
Portable chest X-ray, supine.

His labs were remarkable for an elevated lactic acid 3.4 millimoles/liter, elevated troponin 1.01 nanograms/milliliter, creatinine 1.84 milligrams/deciliter, a white blood cell count of 16.2 k/microliter, and a beta-natriuretic peptide level of 443 picograms/milliliter. Cardiac troponins and beta-natriuretic peptide remained persistently elevated on repeat lab work. Other nonspecific markers of inflammation were elevated including erythrocyte sedimentation rate 90 millimeters/hour, C-reactive protein 2.2 milligrams/deciliter, and ferritin 931 nanograms/deciliter. Electrocardiogram demonstrated tachycardia and T-wave inversions in leads V4, V5, and V6. Chest X-ray showed bilateral interstitial infiltrates which prompted initiation of broad-spectrum antibiotics. Noninvasive positive pressure ventilation was initially attempted without success. The patient subsequently required mechanical ventilation secondary to worsening hypoxemic respiratory failure as evidenced by a low partial pressure of oxygen on arterial blood gas monitoring (Figure 1). Ventilator mechanics yielded a plateau pressure of 27 mmHg with an applied positive end-expiratory pressure of 15 centimeters of water. Bronchoalveolar lavage (BAL) was positive for respiratory syncytial virus, subtype A.

Given his progressive hypotension and impending cardiogenic shock, the patient underwent emergent cardiac catheterization yielding nonobstructive coronary disease. A pulmonary artery catheter was placed with a mean pulmonary artery pressure of 38 mm/Hg and wedge pressure documented at 28 mm/Hg. The patient's pulmonary hypertension was thought to be secondary to elevated left heart pressures given the elevated wedge pressure. He required maximum vasopressor support with norepinephrine, vasopressin, dopamine, and milrinone. An intra-aortic balloon pump was inserted for refractory shock. Viral myocarditis was considered the leading diagnosis at this point given his clinical presentation and worsening cardiopulmonary status.

**Figure 2:**
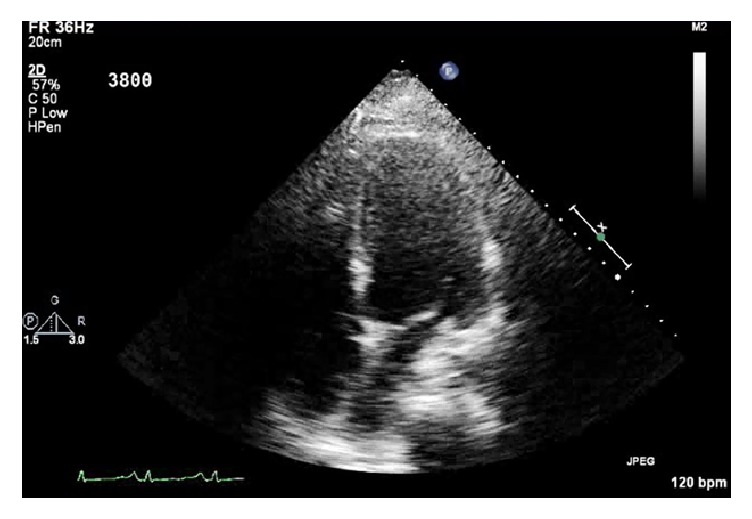
Transthoracic echocardiogram, apical four-chamber view.

Due to his progressive cardiopulmonary failure, the patient required venoarterial extracorporeal membrane oxygenation (VA-ECMO). An intraoperative transesophageal echocardiogram was performed which showed normal size and function of the right ventricle and severely depressed left ventricular systolic function. These findings were consistent with a transthoracic echocardiogram, which showed an estimated ejection fraction of 10–15% (Figure 2). Given his instability, the patient underwent central cannulation for VA-ECMO with left ventricular vent for left heart decompression.

**Figure 3:**
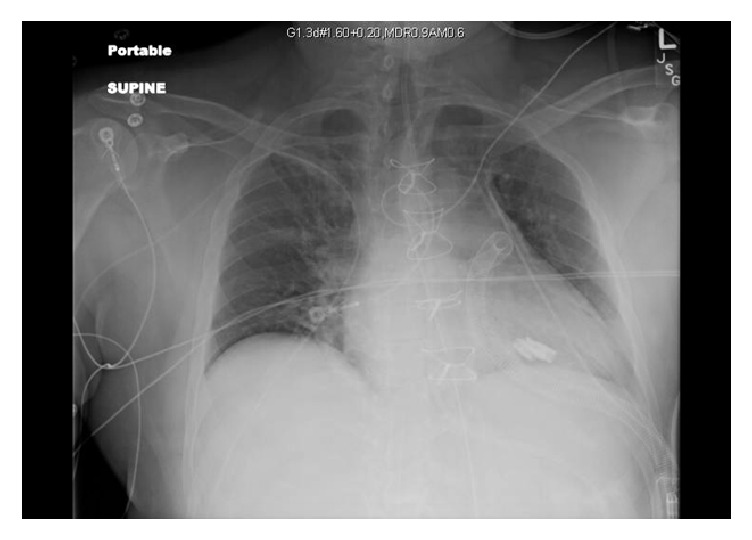
Portable chest X-ray, supine.

On postoperative day one, the patient showed marked improvement in his hemodynamics with minimal vasopressor support needed. The intra-aortic balloon pump was removed and the patient was liberated from mechanical ventilation on postoperative day two. Follow-up chest X-ray showed improvement in the interstitial edema pattern previously noted on prior imaging (Figure 3). Given his excellent lung compliance and rapid resolution of pulmonary pathology, cardiogenic pulmonary edema from decompensated left sided heart failure was favored over acute respiratory distress syndrome.

**Figure 4:**
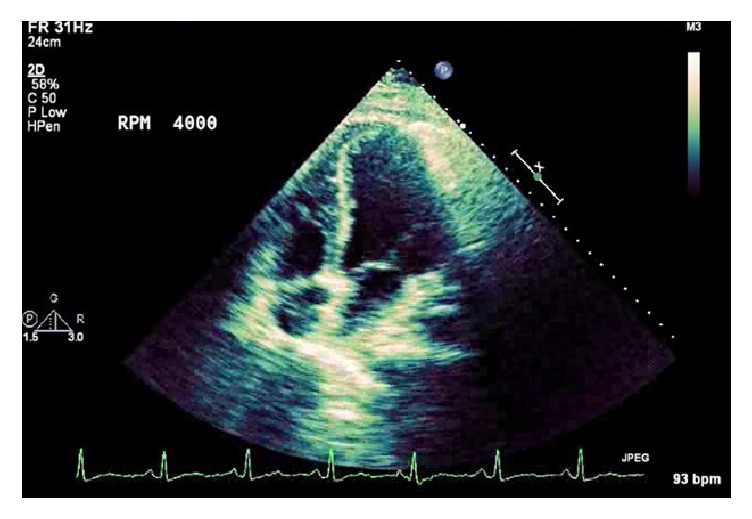
Transthoracic echocardiogram, apical four-chamber view.

**Figure 5:**
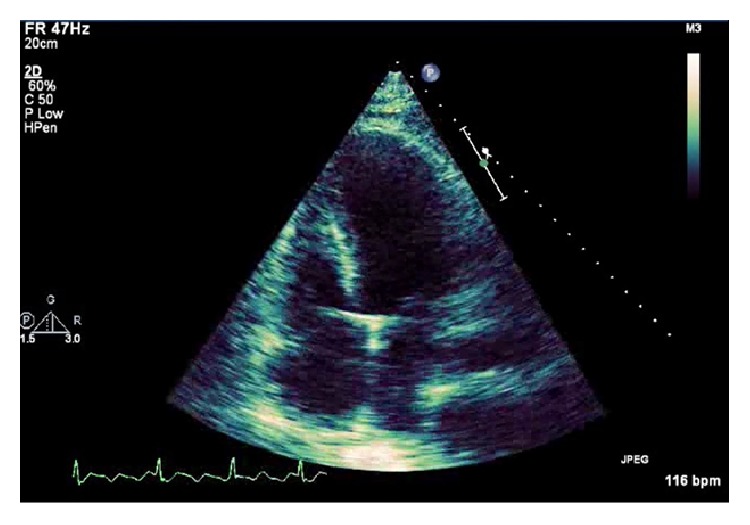
Transthoracic echocardiogram, apical four-chamber view.

Transthoracic echocardiogram performed seven days after admission demonstrated continued reduction in systolic function with an ejection fraction of 20–25% (Figure 4). As the patient improved clinically, explantation from ECMO occurred 20 days postoperatively. Follow-up echocardiogram showed improvement in his ejection fraction to 35–40% (Figure 5). Repeat cardiac biomarkers trended downward and were within normal limits at this time indicating myocardial recovery. The patient was subsequently discharged and was doing well on outpatient follow-up and was compliant with his heart failure medication regimen. Due to the rapid resolution of his pulmonary pathology, it was believed that the duration of VA-ECMO served as a bridge to recovery from cytokine-induced myocardial inflammation secondary to RSV as exemplified by serial imaging.

## 3. Discussion

Myocarditis is an inflammatory cardiomyopathy that may present with new onset cardiac dysfunction ranging from subclinical to cardiogenic shock. Typical symptoms include dyspnea, chest pain, palpitations, and fatigue; however a variety of cardiac abnormalities such as new onset arrhythmias or heart block may also occur. Evaluation includes cardiac biomarkers, electrocardiogram, and echocardiogram. Though endomyocardial biopsy is the gold standard for the diagnosis of myocarditis, newer modalities are being explored due to the technical difficulties and inherent risks associated with this procedure. Cardiac magnetic resonance imaging is noninvasive but has the ability to identify areas of myocardial inflammation.

The major causes of myocarditis include viral and bacterial infections, systemic autoimmune diseases, and various toxins. Treatment typically focuses on alleviation of heart failure symptoms with medication and supportive care. Trials for various immunotherapies are ongoing, but these medications have not yet been recommended for routine use. For patients with progressive disease refractory to medical therapy, circulatory support can be provided with left ventricular assist devices [1, 4–6].

Our patient initially presented with shortness of breath which rapidly progressed to acute hypoxemic respiratory failure due to cardiogenic shock with evidence of myocarditis. Cardiac biomarkers remained elevated throughout his hospital course. Beta-natriuretic peptide was used as a marker for the patient's new onset heart failure and was trended until the time of discharge when values returned to normal range (Figure 6). Since the patient did not have extensive comorbidities and was relatively young, a thorough workup was done to identify the cause of his acute heart failure. He had a history of diabetes mellitus, hypertension, and hyperlipidemia all of which were appropriately managed in the outpatient setting. Echocardiogram and coronary angiogram did not show evidence of valvular lesions or significant coronary disease, respectively, mitigating the possibility of prior myocardial pathology. A urine toxicology screen was negative and serum alcohol level was zero on admission. With no other obvious causes for our patient's acute heart failure, myocarditis became the most likely etiology.

Further workup for viral and bacterial etiologies yielded widely negative results. There was no serological evidence of systemic autoimmune diseases or toxin-induced myocardial injury. Comprehensive viral panels were all negative except for persistently positive RSV subtype A on RT-PCR. Given the acute fulminant nature of his symptoms, resolution with supportive care, positive viral genome, and lack of other confounding variables, RSV myocarditis became the favored diagnosis. Mechanical support with VA-ECMO precluded further diagnostic testing such as cardiac MRI or myocardial biopsy. While definitive diagnosis of myocarditis can only be made histologically, we feel that the diagnostic information accrued offers convincing evidence toward a RSV mediated cytokine-induced inflammatory cardiomyopathy in this patient with stable underlying comorbidities and excellent performance status.

## 4. Conclusion

There is a paucity of literature describing RSV in correlation with fulminant myocarditis, especially in adults. We not only depict a rare infectious entity but additionally describe the use of VA-ECMO (venoarterial extracorporeal membrane oxygenation) as a bridge to recovery. We stress the importance of considering RSV as a possible cause of rapidly fulminant myocarditis and underscore the need for aggressive treatment with VA-ECMO for refractory cardiogenic shock.

## Figures and Tables

**Figure 6:**